# Language acquisition and speech rhythm patterns: an auditory neuroscience perspective

**DOI:** 10.1098/rsos.211855

**Published:** 2022-07-27

**Authors:** Usha Goswami

**Affiliations:** Centre for Neuroscience in Education, Department of Psychology, University of Cambridge, Cambridge, UK

**Keywords:** rhythm, auditory, neurosciences, perspective, language acquisition

## Abstract

All human infants acquire language, but their brains do not know which language/s to prepare for. This observation suggests that there are fundamental components of the speech signal that contribute to building a language system, and fundamental neural processing mechanisms that use these components, which are shared across languages. Equally, disorders of language acquisition are found across all languages, with the most prevalent being developmental language disorder (approx. 7% prevalence), where oral language comprehension and production is atypical, and developmental dyslexia (approx. 7% prevalence), where written language acquisition is atypical. Recent advances in auditory neuroscience, along with advances in modelling the speech signal from an amplitude modulation (AM, intensity or energy change) perspective, have increased our understanding of both language acquisition and these developmental disorders. Speech rhythm patterns turn out to be fundamental to both sensory and neural linguistic processing. The rhythmic routines typical of childcare in many cultures, the parental practice of singing lullabies to infants, and the ubiquitous presence of BabyTalk (infant-directed speech) all enhance the fundamental AM components that contribute to building a linguistic brain.

## 1. Introduction

Human cultures primarily use spoken language for communication, and so a key task for infants wishing to be part of the community is to acquire this language or (more typically) these languages. All human cultures have also invented music. A fundamental feature of both language and music is rhythmic structure. In music, it is more obvious to the listener that there are different levels of rhythmic patterning nested within the overall beat rate; however, this is also the case in human languages. In essence, speech involves patterns of strong and weak beats that recur periodically in hierarchical structures. This patterning is referred to as metre or prosody and syllable stress. Recent computational modelling suggests that the infant brain initially relies on this rhythmic patterning when building a language system [1]. In this Perspective, new insights into the sensory/neural development of a typically functioning language system and its atypical development in developmental dyslexia (DD) and developmental language disorder (DLD) are reviewed. The focus is on the role of acoustic rhythm in explaining both the acquisition of a language system by the human brain, and the atypical developmental trajectories that can ensue when acoustic rhythm is poorly perceived. The core premise is that recent advances in auditory neuroscience support the view that children's acoustic sensitivity to slow amplitude (intensity or energy) changes in the speech signal, a previously neglected area of research, can provide an integrated account of causal developmental mechanisms [2]. Rhythm processing in child language acquisition is explored here from a multi-disciplinary perspective involving behavioural data, child psychoacoustics, computational modelling of infant- and child-directed speech, and neural imaging.

## 2. Behavioural data: the core developmental role of rhythm

Over three decades ago, cross-language behavioural research showed that newborn infants could discriminate languages from different rhythm classes (stress-timed versus syllable-timed), and it was proposed that rhythm discrimination could be a cross-language precursor of language acquisition [3]. Around the same time, it was demonstrated that infant babble reflected the rhythm patterns of the ambient language. Infants listening to Arabic babbled the rhythms of Arabic, whereas infants listening to French babbled the rhythms of French [4]. Babbling follows the rhythmic timing and stress patterns of natural language prosody: it is a specifically linguistic behaviour. Babies exposed to sign languages go through a developmental stage of ‘babbling’ on their hands [5]. Infants are discovering and producing the most rudimentary structures of the natural language to which they are exposed, and for both spoken and signed languages, some of these rudimentary structures are rhythmic ones.

**Figure 1.**
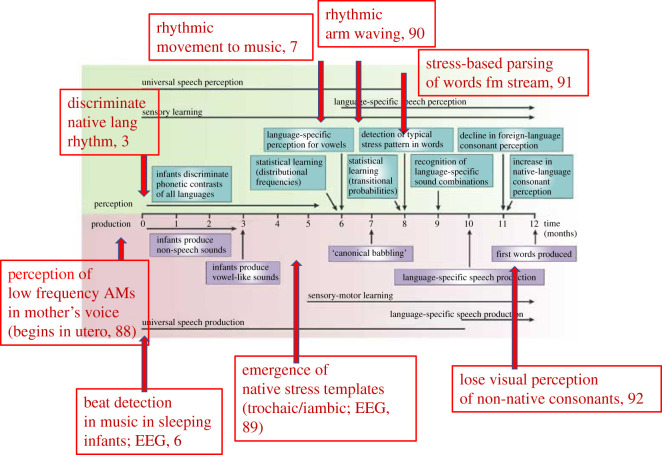
The timeline of infant speech acquisition from Kuhl [9], with perceptual and motor studies that support the rhythmic focus adopted here added in red print. Papers referenced: 88, Hepper & Shahidulla [10]; 6, Winkler *et al*. [6]; 3, Mehler *et al*. [3]; 89, Weber *et al*. [12]; 7, Zentner & Eerola [7]; 90, Iverson [13]; 91, Jusczyk *et al*. [14]; 92, Pons *et al*. [15].

More recently, experimental studies have explored musical behaviours in newborn infants. These studies have revealed sensitivity to different musical beat structures at birth, with systematic rhythmic movement to music measurable by five months of age [6,7]. Indeed, infants tested in Canada are sensitive to rhythm structures in Balkan folk songs which adult North American listeners cannot distinguish [8]. Adult perception is probably shaped by lifelong learning of the rhythmic structures of more Western musical genres. Some of these rhythmic insights from infant behavioural data are shown in figure 1. They are superimposed onto the classic view of infant language acquisition, which is based on the elementary speech sounds thought to make up words, ‘phonemes’ [9]. This view of ‘elementary’ speech sounds is dominant in the infancy field; language acquisition research is dominated by investigations of the sensory learning of phonemes. The idea that spoken words are stored neurally as sequential collections of phonemes ‘akin to a pronouncing dictionary’ has been frequently challenged in speech engineering, because for example the acoustic concatenation of sequences of phonemes did not enable the development of successful speech recognition software [16]. Yet this empirical challenge to the view that phonemes are the basic elements of the neural code for speech has barely impacted the field of language acquisition research.

In contrast to the phoneme-dominant view, a purely acoustic analysis of the speech signal reveals that ‘phonemes’ are an abstraction from the signal itself, and are perceptual categories imposed by the literate listener [17]. If infants do not learn a pronouncing dictionary of phonemes, then what are they learning? The view that they are learning important fundamental acoustic rhythm structures is now gaining ground [18–21]. Many aspects of children's linguistic life, such as children's stories and nursery routines, draw heavily on rhythmic devices to structure language. Knee-bouncing games with infants, nursery rhymes and playground clapping games all depend on the integration of repetitive language and repetitive rhythm. This may not be an accident. Rather, these nursery routines may provide ‘supranormal’ stimuli that support language learning by the human brain [22].

## 3. Behavioural data: atypical perception of rhythm in developmental disorders of language

**Figure 2.**
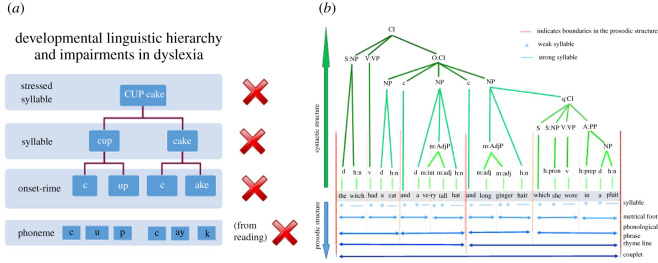
Nested acoustic hierarchies contributing to phonological and syntactic development. (*a*) Schematic depiction of the phonological linguistic hierarchy, conceptualized by linguists as a nested structure. The ability to reflect on sound structures at any level of the hierarchy is impaired in children with DD. (*b*) Illustration of the syntactic and prosodic structure of a line from the children's story ‘Room on the Broom’ (adapted from Richards & Goswami [27]). Abbreviations: d, determiner; h:n, noun, head of noun phrase; v, verb; m:int, modifier:intensifier; m:adj, modifier:adjective; h:pron, pronoun, head of noun phrase; h:prep, preposition, head of prepositional phrase; c, conjunction; q, qualifier; Cl, clause; S, subject; O, object; A, adverb; NP, noun phrase; VP, verb phrase; PP, prepositional phrase.

Disorders of language acquisition carry severe developmental costs. The two most prevalent disorders are DD and DLD (previously called specific language impairment or SLI). Children with DD appear to acquire spoken language with ease, but show difficulties once tuition in reading begins. Despite apparently normal hearing and normal intellectual functioning, they fail to develop age-appropriate reading skills, and these difficulties persist even with intensive remediation [23]. A wealth of behavioural data across languages shows a core difficulty in accessing the phonology (sound structure) of the spoken language [24]. The development of a phonological system, a person's implicit knowledge about the inventory of the sound system of a language, is part of typical pre-school language acquisition, and it is boosted by acquiring literacy [25]. Even prior to literacy tuition, children with DD struggle with phonology. For example, they find it difficult to count the number of syllables in multi-syllabic words like ‘university’ (five syllables), and they find it difficult to hear whether words rhyme. These aural difficulties have led to the behavioural characterization of a ‘core phonological deficit’ in DD across languages [26] (figure 2*a*). Children with DD also have robust and profound difficulties hearing both speech and non-speech acoustic rhythm [28].

Children with DLD have persistent difficulties with learning oral language that cannot be explained by a known condition such as sensori-neural hearing loss, and are typically characterized as having difficulties with the accurate processing and production of grammatical structures in speech [29]. For example, children with DLD may fail to use inflectional endings appropriately (She comb her hair), and may fail to mark tense (Yesterday I fall down). Although the classic characterization of DD versus DLD suggests a neat division regarding phonology (impaired in DD) versus syntax (impaired in DLD), phonological difficulties are not as distinct from grammatical difficulties as the classic analysis suggests and there can be considerable symptomatic overlap [30].

Again, new psychoacoustic understandings may throw light on these developmental patterns. All human languages use prosodic phrasing (tightly integrated hierarchies of metre and syntax) to highlight the grammatical structure of language [27]. This is illustrated in figure 2*b*, using a phrase from the popular children's story book ‘Room on the Broom’, by Julia Donaldson. Accordingly, accurate sensory learning and neural representation of these acoustic rhythmic hierarchies may be crucial to developing syntax and grammar. Children with DLD also have robust and profound difficulties in hearing acoustic rhythm.

The investigation of rhythm perception and production in children with DLD and DD using matched experimental tasks is revealing. The simplest example of a rhythmic behaviour is tapping or clapping in time with a regular beat. Corriveau & Goswami [31] asked children with DLD to tap to a metronome beat. The data showed that the children with DLD were considerably less accurate at synchronizing their taps with the metronome than either age-matched or language-matched (hence younger) control children at rates of 2 Hz and 1.5 Hz. These temporal rates broadly correspond to the inter-stress intervals found in speech [32]. Children with DD show similar profiles. Thomson & Goswami [33] showed that DD children were less accurate at tapping to a metronome beat at 2 Hz and 2.5 Hz compared to age-matched children, and Thomson *et al*. [34] showed impaired tapping in highly remediated adults with DD who were university students. Tapping to a beat is also impaired in children who stutter [35].

On the perceptual side, regarding DLD Cumming *et al*. [36] reported that individual differences in a speech rhythm matching task and a musical beat perception task were significant predictors of children's scores in standardized measures of receptive and expressive language development. Those children with DLD who had more accurate rhythm matching or more accurate musical beat perception had more accurate language scores than those with less accurate rhythmic skills. Similar behavioural data characterize children with DD. Huss *et al*. [37] reported less accurate musical beat perception skills compared to age-matched control children, and Goswami *et al*. [38,39] reported less accurate musical beat perception skills in DD and less accurate speech rhythm perception compared to reading-matched (hence younger) control children. Individual differences in beat perception and speech rhythm perception predicted significant unique variance in phonological and reading tasks.

Using younger controls when studying children with atypical development is important experimentally, to help equate for the effects of learning on the developing brain. In principle, younger language-age-matched or reading-level-matched children provide a way of controlling for the effects of experiencing oral or written language. These younger children have reached the same developmental level of oral or written language learning as the children with DLD or DD. Further, rhythm is a multi-modal percept. Visual rhythm cues given by head and cheek movements synchronized to the act of articulation, as well as motor knowledge of how to prepare the articulators to speak, all converge with acoustic rhythmic information to aid language comprehension [40]. Speech is processed as a sound, as a visual input and as an action. Neurally, rhythm appears to lie at the core of this behavioural convergence, and any or all of these modalities may be affected when development is atypical. For reasons of space, this Perspective focuses primarily on auditory rhythm; the contribution of other modalities to DD and possible sub-types is reviewed in [41].

## 4. Sensory underpinnings: where is rhythm in the speech signal?

**Figure 3.**
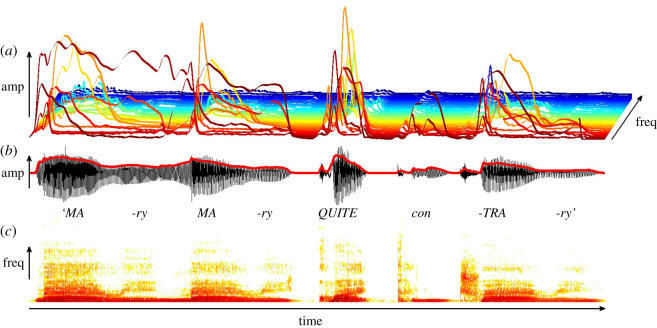
Modelling the structure of the speech signal for the nursery rhyme Mary Mary Quite Contrary. (*a*) A three-dimensional view of the AM structure of speech, with lower spectral frequencies shown in red colours (foreground) and higher spectral frequencies shown in blue colours. The modelling shows that there is most energy in lower frequencies, and energy peaks (AM peaks) coincide with stressed syllables. (*b*) The raw acoustic signal (*x*-axis time, *y*-axis amplitude), and (*c*) the speech signal using the traditional speech spectrogram (*x*-axis time, *y*-axis spectral frequency or pitch). The darker shading in (*c*) represents larger amplitudes. AM, amplitude modulation; AE, amplitude envelope; CDS, child-directed speech. Figure created by Victoria Leong.

Attempts to characterize the acoustic basis of speech rhythm have a long history [42]. Rather than revisit that history here, the focus in this Perspective is on the potential role of the amplitude modulation (AM) structure of the speech envelope in yielding acoustic rhythmic structure. Speech meets the human ear as a sound pressure wave whose shape (amplitude envelope) contains temporal patterns that fluctuate over many different timescales. This AM structure is hidden in the classic depiction of the speech signal used in linguistics, the speech spectrogram (shown in figure 3*c*). The speech spectrogram depicts the raw sound wave (shown in figure 3*b*) in terms of changes in frequency (pitch) over time. The speech envelope contains a range of AM patterns at different temporal rates which are found across the frequencies depicted in the spectrogram, as do many other biological sounds like wind and rain (and as does Western music; see [43]). The dominant AM components of the speech amplitude envelope can also be modelled, as shown in figure 3*a*. Here the frequency range of speech is depicted as a three-dimensional landscape, with the highest frequencies in the far distance. The dominant AM components depicted in the lower frequency regions appear to help infants and children to learn phonology, across human languages [44]. The ability to use these key structural components of the amplitude envelope is particularly important for infants, who must build a phonological system from the speech signal ground-up. It is noteworthy that the cochlear implants that help the deaf brain to build a language system rely on transmitting amplitude envelope information, selecting some of the frequency bands that cover the spectral range of speech (approx. 125–8000 Hz).

**Figure 4.**
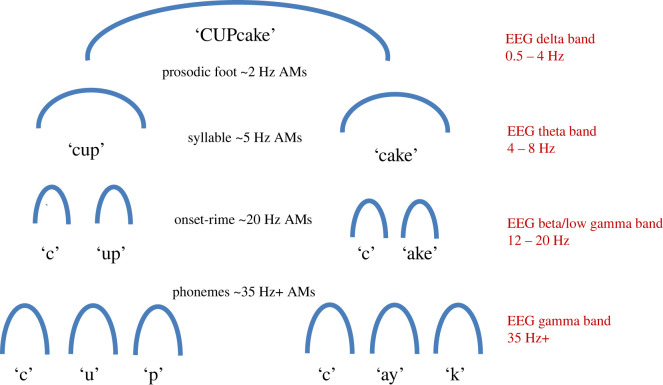
Schematic depiction of the AM phase hierarchy as modelled for English nursery rhymes, showing the nested AM information that supports linguistic parsing of phonological units and the matching brain rhythms. By hypothesis, automatic alignment of neuronal oscillatory networks at matching temporal rates to these nested AMs enables accurate encoding and parsing of the speech signal. This speech–brain alignment appears to be ‘out of time’ for children with language disorders.

The potential role of the acoustic structure of the amplitude envelope in children's linguistic learning can be investigated by separating the AM characteristics of speech from the frequency modulation (FM) characteristics. This is achieved by acoustic engineering methods for decomposing the amplitude envelope (demodulation) [45]. The AM patterns in the speech signal are in essence associated with fluctuations in loudness or sound intensity, while the FM patterns can be interpreted as fluctuations in pitch and noise [45]. Both infant-directed speech (IDS) and rhythmic child-directed speech (CDS, children's nursery rhymes) can be modelled using demodulation approaches [1,46]. The modelling (figure 3*a*) reveals the core spectro-temporal modulation patterning nested in the speech envelope in each speech genre. To achieve this, the speech signal is filtered into the same bands imposed by the human cochlea, and modulation structure is then estimated using a principal components analysis approach. This reveals three core AM bands in both IDS and CDS spanning five core pitch bandings. Modulation structure is computed using key modulation statistics such as AM maxima (peaks in bands) and oscillatory phase relationships (rhythmic synchronicity) between different bands of AMs. This ‘spectral-amplitude modulation phase hierarchy’ (S-AMPH) modelling approach reveals that rhythmic patterning in IDS and CDS is represented in the phase relations of slower AM bands in the amplitude envelope, particularly those corresponding temporally to ‘brain waves’ in the delta (∼2 Hz) and theta (∼5 Hz) bands in electroencephalography (EEG). EEG records brain electrical activity, measuring the intrinsic rhythms of cell signalling in the brain, and the same rhythms (delta, theta, alpha, beta, gamma) are found across the cortex. The acoustic AM phase synchronization for AM bands centred on approximately 2 Hz and approximately 5 Hz in IDS is significantly stronger in IDS than in speech directed to adults, and these AM bands are automatically encoded by brain waves in the infant brain [47]. Further, the acoustic modelling reveals more modulation energy in the ‘delta band’ of AMs in these infant and child speech genres compared to adult-directed speech (ADS). The modulation peak in IDS is approximately 2 Hz, in contrast to the modulation peak in ADS, which is approximately 5 Hz (across languages) [1,48]. Interestingly, this same 2 Hz modulation peak is found in music [43,48]. The most dominant phase relations between AM bands in ADS also differ. ADS has significantly stronger phase synchronization in faster AM bands compared to IDS (these faster bands correspond to the EEG rhythms of theta, ∼5 Hz, and beta/low gamma, ∼20 Hz). Accordingly, words and phrases do not contain equal energy across all modulation rates (timescales) nested in the speech amplitude envelope, and furthermore the speech energy distributions are different in IDS and CDS compared to ADS. Experimental work shows that the phase relations of the ‘delta’ (∼2 Hz) and ‘theta’ (∼5 Hz) AM bands in nursery rhymes are critical to perceiving metrical structure (e.g. trochaic versus iambic) [1,49]. Another core acoustic feature related to speech rhythm is the ‘amplitude rise time’ of the vowel in any syllable [50]. As a syllable is produced, the speech energy or amplitude rises, peaking at the vowel and then falling again. These amplitude rise times seem to function perceptually as auditory temporal ‘edges’, providing clues to structure in a similar way to edges and bars in the visual field. Mechanistically, the beginnings of new linguistic units can be identified via prominent rise times [51]. Not all local rises in amplitude correspond to the onsets of whole syllables, but the onsets of successive syllable-related modulations in the amplitude envelope and their rates of change (rise times) are critical linguistic perceptual events [52]. These rise times vary with the phonetic properties of the syllable (e.g. plosive versus liquid, as in ‘pit’ versus ‘lit’) and are larger when a syllable is stressed [51] (most English nouns begin with a stressed syllable, as in ‘daddy’, ‘mummy’, ‘baby’). The perceptual discrimination of different rise times occurring at different modulation rates across different frequencies in speech is thus vital for extracting linguistically relevant information from the speech signal. Computational modelling using the S-AMPH approach shows that it is even possible to map acoustic structure in the amplitude envelope to linguistic phonological structure at the prosodic, syllable and ‘onset-rime’ levels [46] (to divide a syllable into onset-rime units, linguists divide at the vowel: ‘d-amp’, ‘cl-amp’, ‘st-amp’). In terms of parsing the speech signal into discrete phonological units like syllable stress patterns (called prosodic feet in linguistics), syllables and onset-rime units, the modelling shows that if an AM cycle at a particular temporal rate is assumed to match a particular speech unit, then application of the S-AMPH to English nursery rhyme corpora identifies 72% of stressed syllables correctly, 82% of syllables correctly, and 78% of onset-rime units correctly [46]. If the nursery rhymes are chanted to a regular 2 Hz beat (a temporal rate also prevalent in music, 120 beats per minute, bpm), then the model identifies over 90% of each type of linguistic unit correctly. This 2 Hz rate is thought-provoking, as analyses of the lullabies sung by mothers to their infants across cultures reveal a beat rate of 120 bpm (2 Hz; see [53]). Cross-cultural convergence on this beat rate is consistent with the idea that cultural practices like BabyTalk and lullabies facilitate the infant's automatic extraction of the AM phase hierarchy via sensory learning, beginning in the cradle. In terms of bands of AM, the modelling shows that the approximately 2 Hz AM band (matching the delta brain rhythm) sits at the top of the hierarchy, governing phase relations with faster modulation rates. This is depicted in figure 4.

## 5. Extracting rhythm from the speech signal in DD and DLD: sensory data

In the last 20 years, the potential role of amplitude envelope perception in the ‘phonological deficit’ characterizing dyslexia across languages has been investigated intensively by myself and my colleagues (our studies encompass English, French, Spanish, Hungarian, Chinese and Finnish, thus both ‘stress-timed’ and ‘syllable-timed’ languages; e.g. [18,54–57]). Converging data come from independent groups working in Dutch and Spanish (e.g. [58–61]). The typical approach in these studies has been to use psychoacoustic tasks that measure the just-noticeable-difference in rise time or acoustic edge detection for a child, and to use non-speech stimuli (sine tones or speech-weighted noise). These studies show robust discrimination impairments in children with DD compared to both their same-age peers and to younger reading-level-matched control children [38]. Studies of children and infants at family risk for dyslexia augment these data by showing impaired rise time discrimination at 10 months in at-risk infants [62]. By their second year, the at-risk infants showed impaired phonological learning of new words along with delayed achievement of phonological constancy (recognizing the same word produced by different speakers; see [63,64]). Rise time detection in infancy showed significant longitudinal associations with vocabulary size at age 3 years [65], and by age 4 non-speech rhythm deficits were present in the at-risk group (using the same musical beat perception task used by Huss *et al*. [37]; see [66]. In studies with at-risk pre-schoolers conducted in English and Dutch, rise time measured in pre-schoolers aged 3–5 years predicted phonological awareness, letter knowledge and reading at ages 6 and 7 years [67,68].

Other studies have assessed sensitivity to AM directly by using temporal modulation transfer functions (TMTFs). TMTFs assess the minimum depth of AM (variation in intensity) that can be detected by a listener. Studies with French DD children demonstrate reduced discrimination of AM at a range of temporal rates, with the largest difference at 4 Hz [69,70]. English-speaking adults with DD also show reduced AM discrimination across a range of temporal rates [71]. Accordingly, both discrimination of amplitude rise times and of AM is impaired in participants with DD, and for rise time this difference is measurable in infancy. These data suggest that children at risk for DD are encoding poorer-quality representations of the speech signal from the get-go, in part via impaired automatic (statistical) sensory learning of the AM phase hierarchy nested in the speech envelope. As noted, it is this hierarchy that helps to support the extraction of phonological units at different linguistic grain sizes (prosodic feet, syllables, onset-rimes) [46].

Amplitude rise time discrimination is also impaired in children with DLD, although to date only English-speaking children have been tested [72–74]. Although not yet studied, it seems likely that poor rise time discrimination would impair extraction of the acoustic prosodic hierarchies that underpin syntactic phrasing (figure 2*b*), as these are also AM hierarchies [27]. The perception of AM information in the speech envelope can be studied in children by using noise-vocoded speech. Noise-vocoded speech is natural speech that has been degraded to increase the perceptual reliance on speech envelope cues [75]. Johnson and colleagues found that children with disorders of ‘speech-sound processing’, essentially similar to children with DD or with both DD and DLD, were significantly impaired in recognizing sentences presented as noise-vocoded speech compared to typically developing age-matched control children. The children with both diagnoses (DLD + DD) showed the greatest sensory impairments.

## 6. Extracting rhythm from the speech signal in DD: neural data

Recent data from auditory neuroscience studies with adults show that rise times/auditory edges also have an important *neural* function regarding encoding the speech signal. Speech encoding relies in part on matching the rhythmic changes in electrical brain potentials in large cortical cell networks (neuronal or neuroelectric ‘oscillations’) to temporally matching rhythmic energy modulations (AMs) in speech. The functional organization of the bands of neuroelectric oscillations responsive to speech inputs (primarily the brain rhythms at delta (0.5–4 Hz), theta (4–8 Hz), beta (12–20 Hz) and gamma (35+ Hz)) is also hierarchical [76], and the temporal rates of oscillation of these brain rhythms match the AM rates nested in the speech amplitude envelope [77]. Rise times/acoustic edges function as sensory landmarks that automatically trigger brain rhythms and speech rhythms into temporal alignment. This occurs via the acoustic ‘edges’ phase-resetting ongoing neuronal oscillations [78,79] (but see also [80]). Successful phase resetting is known to improve speech intelligibility for adults [81]. Accordingly, a nascent language system can in principle be extracted from the speech signal via the *automatic* alignment of neuroelectric oscillations to the AM information hierarchy in speech [2] via the automatic detection of rise times. Extracting ‘acoustic-emergent’ phonological and syntactic information via automatic statistical learning likely requires extremely accurate speech–brain alignment.

Studies of this speech–brain alignment in children with DD suggest that the alignment process is disrupted in dyslexia, particularly in the delta band (0.5–4 Hz). When listening to noise-vocoded sentences, the accuracy of speech–brain oscillatory alignment in the delta band (0–2 Hz) was found to be significantly reduced for DD children compared to *both* younger reading-level controls and chronological age-matched controls, even when sentence recognition accuracy was matched across groups [82]. Despite equivalent perceptual recognition (tested by repeating back the sentences, matched across groups), the dyslexic brain showed less accurate encoding of speech envelope information at approximately 2 Hz. This suggests that the DD children were relying on other speech-based information to complete the task. Atypical delta-band speech encoding has also been reported using a passive story listening task by Di Liberto *et al*. [83]. Children with DD showed quite different scalp patterns regarding speech–brain alignment compared to both reading-level and age-matched controls, with the largest group differences found in the right hemisphere. Furthermore, in rhythmic language processing tasks (e.g. listening to syllables like ‘ba’ repeated every 500 ms), the dyslexic brain shows a reliable phase difference in delta-band speech–brain alignment compared to age-matched-control children [84,85]. This difference is found even when the acoustic threshold for hearing when an oddball syllable breaks the rhythm is equated between groups. The phase shift is very small (approx. 12.8 ms; see [84]). Nevertheless, even this small disruption in speech–brain alignment means that the dyslexic brain is ‘out of time’, negatively affecting perception of the entire AM hierarchy (due to its phase-dependent nature). If one band of AMs is ‘out of time’, this would affect phase relations between the other AM bands. Important speech information would arrive out of time for the DD brain, hence the perceptual experience of the entire speech signal would be subtly different. Atypical speech–brain alignment would then presumably impair the extraction of phonology. Via the S-AMPH modelling, we can expect that the automatic extraction of the AM phase hierarchy and consequently the development of acoustic-emergent phonology would be negatively affected.

Regarding rhythm production, if children with DD are trained to tap to every second pulse of a 2.4 Hz metronome beat so that their synchronization performance is no less variable than control children, their brains still show a phase difference at 2.4 Hz when EEG is recorded [86]. This phase difference is also present during passive auditory listening to the beat. Again, the data suggest that the dyslexic brain is ‘out of time’ regarding alignment in the delta band, and that this asynchrony is related to the typically greater variability of DD children's tapping. Simple tapping tasks may thus provide a good index of the accuracy of speech–brain alignment in children [22].

EEG studies with highly remediated adults with DD have also been conducted. In a study when a rhythmic tone stream was delivered at 2 Hz, phase entrainment was significantly reduced in DD compared to control adults [87]. The participants were required to press a button whenever white noise replaced the repeating tones, and the adults with DD were matched for speed and accuracy to the control adults in the button-press paradigm. Yet whereas the control participants showed faster responses in the *rising phase* of the delta oscillation, which would be expected if sensory performance is enhanced at the oscillatory peak (more neurons are active at the peak), the DD participants showed no relationship between oscillatory phase and behaviour. This suggests that reaction time in the DD adults was not governed by oscillatory phase in delta, and hence that they were using other sensory strategies to succeed in the task. Finally, although cross-language neural data are sparse, the sentence listening task has also been used with Spanish children with DD [88]. The authors reported impaired oscillatory alignment to speech in the delta band, with reduced delta synchronization originating in right primary auditory cortex [88]. This is important, as according to linguistic analyses Spanish has a syllable-timed rhythm structure while English has a stress-timed rhythm structure. The neural data suggest that the brains of children with DD learning both syllable-timed and stress-timed languages are encoding a significantly less accurate representation of low-frequency (delta band) envelope information in the speech signal related to extracting prosodic structure [46]. This isolates a potentially causal neural locus of impairment that could be targeted for remediation. Similar studies with children with DLD are yet to be conducted.

## 7. ‘Temporal sampling’ theory of language acquisition, DD and DLD

To capture these diverse data from psychoacoustic, neural, behavioural and speech modelling studies, a ‘temporal sampling’ (TS) framework for language acquisition can be proposed [2,19,22,28]. The successive refinements of TS theory aim to explain infant and child data regarding rhythm, phonology, syntax, sensory processing of rise time/AMs and language development, although TS theory was originally proposed to explain only phonological difficulties in dyslexia [19]. TS theory is based on the fact that the brain processes the sensory world in a series of snapshots. Cell networks ‘sample’ sensory input in parallel in different temporal integration windows and then bind their outputs together to create a seamless sensory world. TS theory proposes that the automatic alignment of endogenous brain rhythms with rhythm patterns in speech is critical for linguistic and phonological development, and that this unconscious alignment (or sampling) process is atypical regarding low-frequency envelope information for children with DD and DLD from birth [2,19,22,28]. This is thought to occur in part because the sensory (acoustic edge or rise time) cues that trigger automatic neural alignment are perceived poorly in DD and DLD. Accordingly, the perceptual organization of speech information (assigning acoustic elements to the groupings comprising linguistic units, and perhaps assigning motor and visual rhythmic elements also) is atypical. For example, if the perceptual organization of AMs is temporally different, then stressed syllables, syllables and the onset-rime units in words would be poorly encoded. Consequently, the development of linguistic processing would be atypical, which would lead to impaired phonological and/or syntactic learning for affected children.

**Figure 5.**
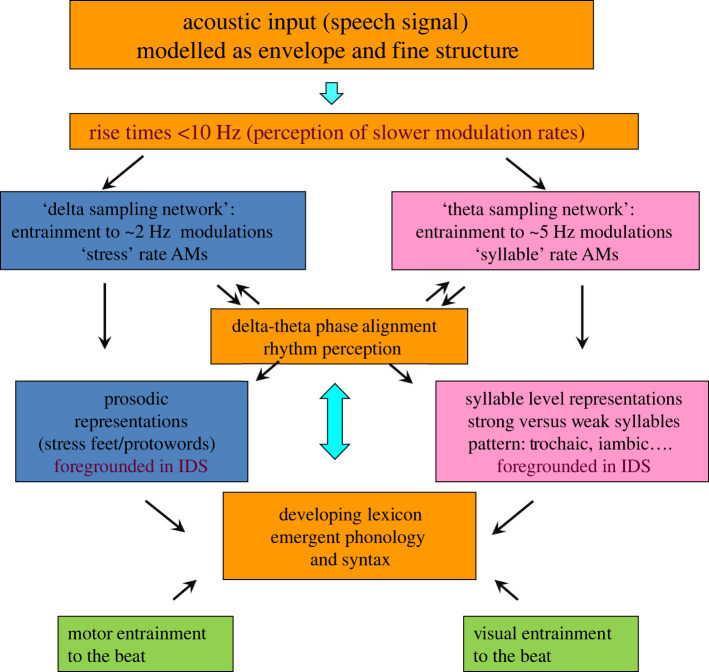
TS for infant language acquisition. Adaptation of TS theory [19] for infants. The schematic depiction emphasizes the core role of rhythm processing via automatic neural entrainment to amplitude modulations at approximately 2 Hz (delta) and approximately 5 Hz (theta) rates and delta–theta phase alignment.

A possible theoretical instantiation of TS theory is provided as figure 5. As noted earlier, when we speak, we are creating sound waves, moving energy through the air. The brain waves record these energy changes, which relate to speech rhythms. The brain does this in part by aligning intrinsic brain rhythms to the AM-driven rhythm patterns in speech. Metaphorically, our brain waves are *surfing* the sound waves. For a successful surfer, the temporal accuracy and prediction of this alignment is key to catching the peak of the wave. TS theory proposes that the brains of typically developing infant surfers make accurate judgements about the peaks of these waves, but that the brains of infants at family risk for DD and DLD do not. This alignment process is automatic; it is part of language acquisition for every baby, but for infants with less accurate rise time discrimination, the automatic alignment is out of time at one or more temporal rates (delta and theta for DD, and possibly slower timescales related to perceiving prosodic phrasing for DLD, these slower timescales remain to be investigated neurally). *Oral* language processing is atypical in both DD and DLD because these automatic brain processes are working slightly differently, although this is easier to demonstrate experimentally in perception than in production. Oral timing studies with DD adults enable the expected speech production differences to be calibrated [89]; to date similar studies with children with DD and DLD are lacking. Nevertheless, toddlers at family risk for dyslexia are known to take longer with articulatory planning than other toddlers, engaging in shorter speaking turns and using fewer syllables [90], and many children with DLD are ‘late talkers’.

Indeed, although children with DD appear to comprehend and produce spoken language without notable differences from other children, closer investigation shows that this is not the case. Their performance in phonological awareness tasks and their word finding difficulties provide experimental examples [24]. Perceiving some of the energy changes in the speech signal differently from other children can be compared to being colour blind. If you are colour blind, you can still see and you can still navigate your environment, but your sensitivity to certain wavelengths of light is reduced. You cannot really distinguish reds, greens, browns and oranges, they look very similar. So if you were learning something at school where you were continually forced to make red/green distinctions, you would be struggling. You would be trying as hard as the other children, but somehow the whole process would be a much bigger effort. TS theory proposes that in dyslexia, children can still hear and can still learn language, but they cannot really distinguish speech rhythm patterns. Stressed and unstressed syllables sound very similar. So when they have to process speech written down, that is to read, it is a big effort. The letter sounds do not seem to add up to words (they do not match the child's linguistic structures) in an easily recognizable way. Reading is always a big effort, even when a child has learned by heart the individual letter–sound correspondences or the individual characters in Kanji or Devanagari or the other visual symbols that comprise the written form of the language. TS theory argues that reading is always a big effort because the dyslexic brain encodes the sound structure of language (phonology) in a subtly different way from the brains of the people who invented a culture's writing systems.

By contrast, children with DLD clearly do not comprehend and produce language as other children do. Their difficulties in perceiving some of the energy changes in the speech signal [72–74] lead to overt behavioural difficulties. Even comprehending spoken language is a struggle. TS theory proposes that it is a struggle because the DLD brain encodes the prosodic phrasing hierarchies in spoken language differently (see [27] for a theoretical overview; note that neural data are still lacking). Children with DLD have documented difficulties in perceiving rhythm and metre [27,91]. They are less skilled at using prosodic phrasing to work out where the verb in a sentence is likely to occur, or to predict the placement of the closure of a syntactic phrase [27]. Behavioural measures of their prosodic perception, such as stress matching tasks, and oral stress misperception measures (which is correct, LA-dybird or lady-BIRD) are related to standardized measures of their language processing [92]. As these children develop, their language difficulties begin to impact on many other areas of cognitive development and the curriculum, leading to poorer educational outcomes and a higher risk of mental health disorders. Accordingly, evaluating simple rhythmic indicators of those at risk could be very important [20,93].

Finally, it may be possible to adapt the speech signal in light of our discoveries about the AM hierarchy, so that the amplitude envelope cues in speech that are perceived poorly by children with DD and DLD are synthetically amplified or exaggerated. Envelope enhancement algorithms are already being applied in DD, with measurable benefits for hearing speech-in-noise [11,94]. A clear prediction from the studies discussed in this Perspective would be that receiving adapted speech from a young age should improve the language processing skills of children at family risk for DLD and DD, possibly enabling their language systems to develop age-appropriately. Although technically complex, such adaptations are within the reach of current technology. For example, some of the processing done by the electrodes in cochlear implants have the effect of enhancing the amplitude envelope of speech. We take it for granted that children who are born short-sighted can be fitted with glasses. Within the next decade, it is possible that children who are born at risk for language impairments may be offered a listening device that lessens the impact of the sensory/neural processes that contribute to DD and DLD, before risk translates into a major disorder of language acquisition.

## Data Availability

This article has no additional data.
